# 3-Hydroxy Kynurenine Treatment Controls *T. cruzi* Replication and the Inflammatory Pathology Preventing the Clinical Symptoms of Chronic Chagas Disease

**DOI:** 10.1371/journal.pone.0026550

**Published:** 2011-10-19

**Authors:** Carolina P. Knubel, Fernando F. Martínez, Eva V. Acosta Rodríguez, Andrés Altamirano, Héctor W. Rivarola, Cintia Diaz Luján, Ricardo E. Fretes, Laura Cervi, Claudia C. Motrán

**Affiliations:** 1 Departamento de Bioquímica Clínica, Facultad de Ciencias Químicas, Centro de Investigaciones en Bioquímica Clínica e Inmunología (CIBICI-CONICET), Universidad Nacional de Córdoba, Ciudad Universitaria, Córdoba, Argentina; 2 Cátedra de Física Biomédica, Facultad de Ciencias Médicas, Universidad Nacional de Córdoba, Santa Rosa, Córdoba, Argentina; 3 Facultad de Medicina, Instituto de Biología Celular, Universidad Nacional de Córdoba, Córdoba, Argentina; National Council of Sciences (CONICET), Argentina

## Abstract

**Background:**

3-Hydroxy Kynurenine (3-HK) administration during the acute phase of *Trypanosoma. cruzi* infection decreases the parasitemia of lethally infected mice and improves their survival. However, due to the fact that the treatment with 3-HK is unable to eradicate the parasite, together with the known proapoptotic and immunoregulatory properties of 3-HK and their downstream catabolites, it is possible that the 3-HK treatment is effective during the acute phase of the infection by controlling the parasite replication, but at the same time suppressed the protective T cell response before pathogen clearance worsening the chronic phase of the infection. Therefore, in the present study, we investigated the effect of 3-HK treatment on the development of chronic Chagas’ disease.

**Principal Findings:**

In the present study, we treated mice infected with *T. cruzi* with 3-HK at day five post infection during 5 consecutive days and investigated the effect of this treatment on the development of chronic Chagas disease. Cardiac functional (electrocardiogram) and histopathological studies were done at 60 dpi. 3-HK treatment markedly reduced the incidence and the severity of the electrocardiogram alterations and the inflammatory infiltrates and fibrosis in heart and skeletal muscle. 3-HK treatment modulated the immune response at the acute phase of the infection impairing the Th1- and Th2-type specific response and inducing TGF-β-secreting cells promoting the emergence of regulatory T cells and long-term specific IFN-γ secreting cells. 3-HK *in vitro* induced regulatory phenotype in T cells from *T. cruzi* acutely infected mice.

**Conclusions:**

Our results show that the early 3-HK treatment was effective in reducing the cardiac lesions as well as altering the pattern of the immune response in experimental Chagas’ disease. Thus, we propose 3-HK as a novel therapeutic treatment able to control both the parasite replication and the inflammatory response.

## Introduction

Chagas disease, caused by the obligate intracellular hemoflagellate protozoan parasite *Trypanosoma cruzi*, is an endemic disorder affecting 17 million people and remains an important public health problem in Latin America [1]. The acute phase of the disease is characterized by a large parasite replication with the trypomastigotes (Tps) present in the blood of infected people several years after the primary infection, and the infection can be transmitted by infected blood transfusion and organ transplant from donors originating from areas of Latin America where the disease is endemic. This situation has transformed Chagas disease into a worldwide concern that could have severe consequences for human health over the long term [2].

In the mammalian host, the parasite’s biological cycle includes the nondividing, blood-circulating Tps, which infect the nucleated cells and also the replicating intracellular amastigotes (Am) that reside in the cytoplasm of the infected cell [3]. The human pathology is extremely diverse and depends on the parasite biology as well as its relationship with the host [4]. The chronic phase of the infection frequently involves long-lasting inflammatory lesions and immune system disorders, with progressive pathology in the heart, esophagus, or colon. Although chagasic megaesophagus and megacolon produce these typical clinical conditions in 5% to 10% of patients, Chagas cardiomyopathy is by far the most serious form of the disease [5].

Chronic Chagas heart disease is a slowly evolving inflammatory cardiomyopathy that may lead to severe cardiac dilatation, congestive heart failure and death [6]. The most typical functional heart abnormalities revealed by electrocardiogram (ECG) are intraventricular conduction disturbances (IVCD), sinus bradycardia and arrhythmia, with histological changes including the degeneration of the cardiomyocytes coexisting with fibrosis and mononuclear cell infiltration [7]. The presence of a cardiac inflammatory infiltrate in the apparent absence of parasites suggests that an autoimmune component could be involved in the pathogenesis of the disease [8]. In Chagas disease, the unresolved infection and the inappropriately balanced inflammation could be two important factors that contribute to chronic diseases and to initiate an autoimmune response.

Indoleamine 2,3 dioxigenase (IDO) is an intracellular enzyme which is constitutively expressed in several human and mouse cells. Being present in innate immune cells, such as macrophages and dendritic cells, IDO catalyzes the initial rate-limiting step of the tryptophan (Trp) catabolism, leading to the production of L-kynurenine (Kyn), 3-hydroxykynurenine (3-HK), 3-hydroxyanthranilic acid (3-HAA) and quinolinic acid among others (collectively known as “kynurenines”) [9]. The IDO gene promoter contains multiple response elements to proinflammatory mediators, demonstrating the strong correlation between inflammation and induced IDO expression [10]. Thus, it has been proposed that IDO activity and “kynurenines” help to tame exaggerated inflammatory responses through the inhibition of a T cell proliferation, promotion of T cell anergy or death and the generation of regulatory T (Treg) cells that drive peripheral tolerance [11]–[14]. Moreover, because of its ability to inhibit the proliferation of facultative intracellular pathogens, it is assumed that IDO forms part of the innate host defence against infections [15], [16]. We have demonstrated that IDO activity is up-regulated after *T. cruzi* infection in mice, with the blocking of IDO activity *in vivo* impairing mice resistance to infection and exacerbating the tissue and blood parasite load and the infection associated pathology [17]. In addition, in contrast to the observed for others intracellular pathogens which are sensitive to Trp starvation, we have previously demonstrated that *T. cruzi* Am and Tps are sensitive to the Kyn downstream metabolite 3-HK, and the therapeutic administration of 3-HK (1 mg/kg/day, intraperitoneally) during the acute phase of the infection decreased the parasitemia and improved the survival of lethally infected mice [17], suggesting that the pharmacologic intervention of IDO pathway could be used as a novel antitrypanosomatid therapeutic strategy. Due to the fact we have demonstrated that treatment with 3-HK is unable to eradicate the parasite during the acute phase of the infection, together with the known proapoptotic and immunoregulatory properties of 3-HK and their downstream catabolites, it is possible that although 3-HK treatment may be effective during the acute phase of the infection by controlling the parasite replication, at the same time it suppresses the protective T cell response before pathogen clearance, thus worsening the chronic phase of the infection. On the other hand, another possible effect of 3-HK treatment (highly desirable) could be the restriction of pathogen growth together with the prompt activation of immunoregulatory mechanisms able to control the Chagas disease’s characteristic pathogenic inflammation. In the present study, mice infected with a non-lethal Tps dose able to develop the chronic phase of Chagas disease were treated therapeutically with 3-HK during 5 consecutive days and the effect of this treatment on the development of chronic Chagas disease was investigated. Our results show that the early 3-HK treatment was effective in reducing the cardiac lesions as well as altering the pattern of the immune response in experimental Chagas’ disease. Thus, we propose 3-HK as a novel therapeutic treatment able to control both the parasite replication and the inflammatory response.

## Results

### 3-HK treatment of acutely *T cruzi*–infected BALB/c mice markedly reduces the severity of chronic Chagas disease

**Figure 1:**
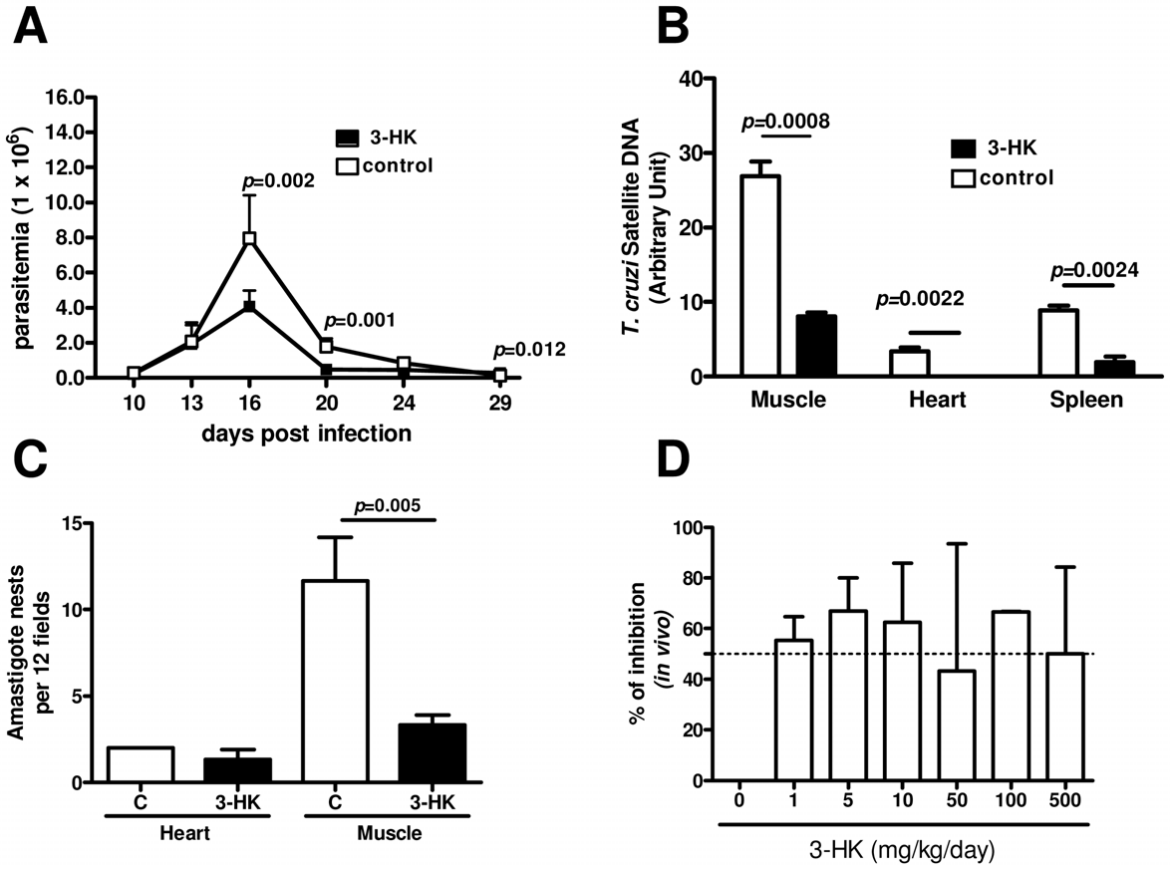
3-HK treatment controls the parasite load in blood and target tissues. Parasitemia, histological analysis, relative amount of parasite DNA and ED_50_ were determined in 500-Tps infected mice treated with 1 mg/kg/day of 3-HK (3-HK) or vehicle (control) from dpi 5 to 10 by the i.p. route. (A) Quantitation of parasitemia (Tps/ml blood). Results are means ± SD of 6–8 mice/group. (B) Quantitation of the number of nests of amastigotes at 3 levels of heart and skeletal muscle from control (*n = *3) and 3-HK (*n* = 4) groups at 16 dpi, employing Axio-Vision 3.0.6 software. (C) Relative amount of *T. cruzi* satellite DNA in the skeletal muscle, heart and spleen from 20-day *T. cruzi* infected 3-HK and control mice. Murine GAPDH was used for normalization. Data are shown as mean ± SD of triplicates, n = 4 mice per group. (D) Parasitemia (Tps/ml blood) at 16 dpi was recorded in 500-Tps infected mice treated with different 3-HK doses (1, 5, 10, 50, 100 and 500 mg/kg/day) or vehicle. The mean value determined for a group of 6–8 mice was used to calculate the percentage of inhibition in parasitemia respect to the vehicle control group. The percentage of inhibition was calculated as [(C-3-HK)/C]×100 where C is the parasitemia in the control group and 3-HK is the parasitemia in the treated group. Results are means ± SD of 6–8 mice/group. One representative of two experiments is shown.

To investigate the role of 3-HK treatment in determining the outcome of chronic Chagas disease, we infected BALB/c mice with 500 Tps of *T. cruzi* and 5 days post infection (dpi) the mice were treated daily with different 3-HK doses or PBS (control) for 5 consecutive days (dpi 5–10). The Tps dose was selected in view of the fact that almost all 500-Tps-infected mice were able to develop the acute infection and progress to the chronic phase. Mice infected with *T. cruzi* and treated with 1 mg/kg/day of 3-HK (3-HK mice) showed lower levels of parasitemia than control mice, but as was observed when a letal *T. cruzi* dose was used [17], no sterilizing effect was observed (Figure 1A). At the peak of parasitemia of control mice (day 16), 3-HK mice presented a significant reduction in circulating parasites, (4.07±0.91×10^6^ *vs.* 7.95±2.44×10^6^ parasites/ml, *p*<0.002) (Figure 1A, C). A histological analysis of hearts and skeletal muscle from 3-HK and control mice on 16 dpi revealed the typical histopathological alterations of acute chagasic inflammation, with the presence of nests of *T. cruzi* Am and foci of lymphomononuclear inflammatory infiltrates (not shown). As was described previously [18], we found more tissue parasitism in skeletal muscle than in cardiac tissue, with 3-HK mice showing significant between-group differences in the skeletal muscle parasite nest number (Figure 1B). In agreement, 3-HK mice showed a significant reduction in tissue parasitism evaluated by real time PCR to detect *T. cruzi* DNA (Figure 1C). On the other hand, the treatment with 3-HK did not adversely affect the normal histology in uninfected mice (NI) (not shown). These results demonstrated that 3-HK treatment of mice infected with *T. cruzi* was able to control the parasite load in blood and target tissues.

To evaluate whether higher 3-HK doses could be effective in eradicating the blood parasites, doses of 5, 10, 50, 100 and 500 mg/kg/day were assayed. As shown in Figure 1D, all administered doses were able to decrease the parasitemic peak by 40 to 65% compared to parasite peaks in the control group. However no significant differences were observed between the assayed doses. For this, we chose the dose of 1 mg/kg/day for this study. Also, we observed that the animals did not show any toxic symptoms during the time of the study, for any of the administered doses.

**Figure 2:**
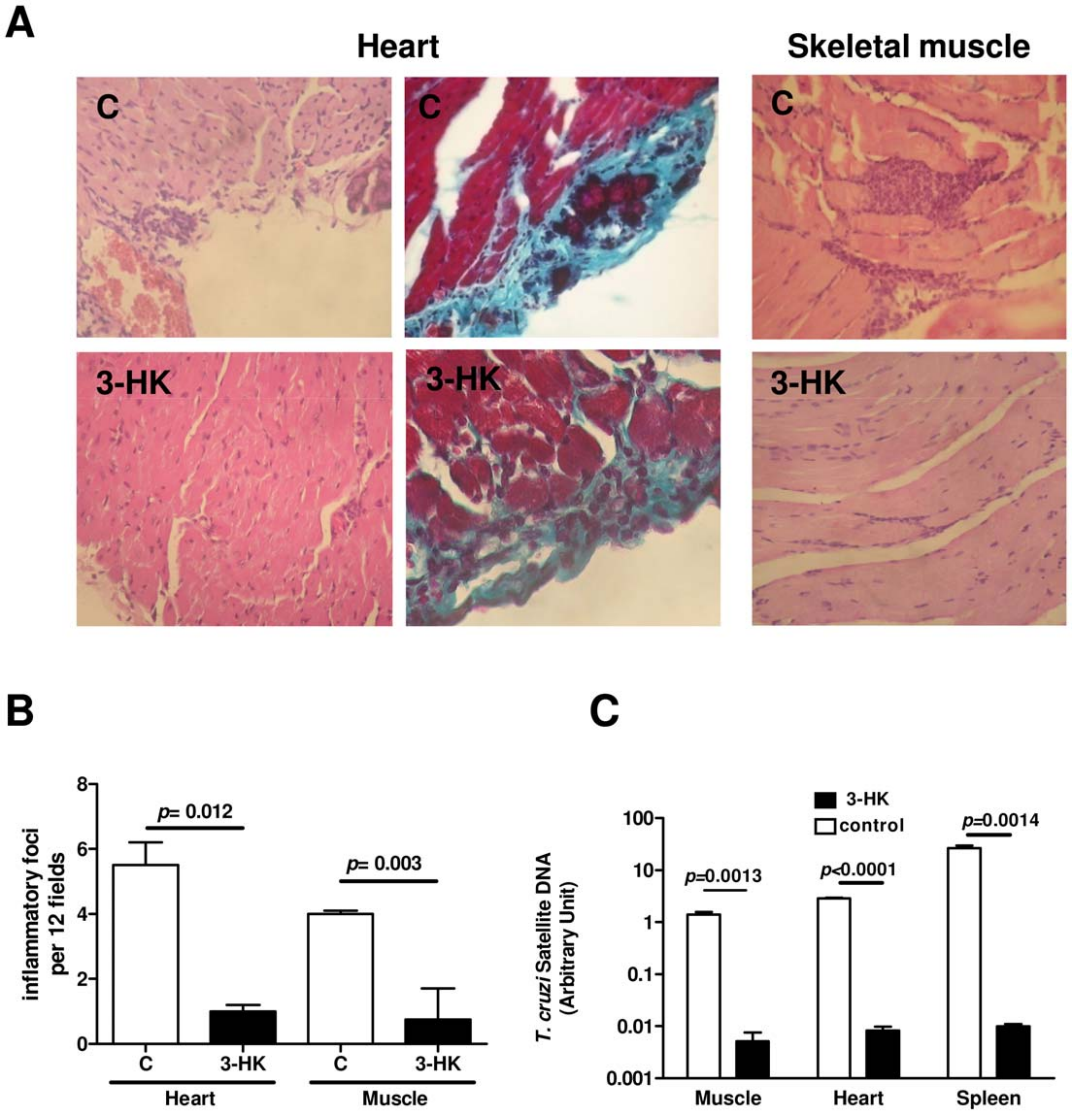
3-HK treatment impairs chronic Chagas’ disease-associated inflammatory pathology. Heart and skeletal muscle sections obtained at dpi 60 from 500-Tps-infected mice, treated (3-HK) or not (C) with 3-HK, were stained with H/E and Masson trichrome staining. (A) Representative histological sections of heart (left panels) and skeletal muscle (right panel) from control (upper panels) and 3-HK mice (lower panels). Left and right panels showing focal mononuclear cell infiltrates stained with H/E. Middle panel showing collagen deposition stained with Masson trichrome stain (X100). (B) Quantitation of foci of cellular in flammatory infiltrates at 3 levels of heart and skeletal muscle from control (n = 4) and 3-HK (n = 4) groups, employing Axio-Vision 3.0.6 software. (C) Relative amount of *T. cruzi* satellite DNA in the skeletal muscle, heart and spleen from 60-day *T. cruzi* infected 3-HK and control mice. Murine GAPDH was used for normalization. Data are shown as mean ± SD of triplicates, n = 4 mice per group.

Sixty dpi (chronic phase), the cardiac electrophysiology was studied by ECG, which revealed a significant decrease in the incidence and the severity of the alterations in 3-HK mice compared with controls (Table 1). Definite ECG abnormalities were found in 75% of the control mice, such as arrhythmias and intraventricular conduction disturbances (IVCD) from mild to severe and heart block, whereas only 20% of the 3-HK mice showed some mild IVCD (*p*<0.01) (Table 1). To evaluate the efficacy of early 3-HK treatment in preventing the development of chronic lesions in *T. cruzi* infected mice, a comparative histological analysis of the inflammation and fibrosis in cardiac tissue and skeletal muscle of infected 3-HK treated and non-treated mice at 60 dpi was performed. Figure 2A and B shows that the treatment with 3-HK led to a reduction in the number of inflammatory foci containing 15 or more cells in skeletal muscle and cardiac tissue. Additionally, 3-HK-treated mice showed less epicardial fibrosis (collagen deposition) and a lack of dystrophic calcification compared with that in non-treated mice (Figure 2A). Moreover, we also confirmed the acute phase findings showing that, even though not clear the infection, 3-HK treatment is effective in reducing the levels of parasite load in the target tissues (Figure 2C).

**Table 1:** Electrocardiographic results in 3-HK treated, Control (infected and untreated) and Normal non-infected (NI) mice at 60 days post-infection.

Animal group	Heart rate (beats/min)	PQ interval (s)	QT interval (s)	Axis (grade)	Mice showing abnormality (%)
3-HK (n = 10)	273.8 (12.8)	0.01–0.04	0.01–0.04	65 (2.2)	20
Control (n = 8)	321.8 (14.3)	0.01–0.02	0.01–0.08	66.2 (2.5)	75*
NI (n = 10)	240 (28.6)	0.02–0.02	0.02–0.06	80 (4.9)	0

Numbers in parentheses are standard errors.

Prolonged QT: intraventricular blockade.

*p <0.01 vs 3-HK or NI using ANOVA and Fisher exact test.

These results indicate that 3-HK treatment plays a critical role in the parasite clearance and control of the inflammatory associated pathology by decreasing the parasite load in blood and tissues and preventing chronic tissue alterations.

### 3-HK treatment modulates the parasite specific immune response during the acute phase of the infection and promotes the development of long-term IFN-γ secreting cells

In our next set of experiments, we investigated whether the 3-HK treatment conditions the development of any particular cellular or humoral immune response able to contribute to the parasite clearance and/or the control of the inflammatory associated pathology.

We analysed the T cell compartment and the cytokine response against parasite antigens at different times pi. No significant differences in the absolute number of total spleen cells were observed between 3-HK and control mice (not shown). However, a significant reduction in the absolute number of CD4+ T cells in spleen from 3-HK mice compared with control mice was observed at 30 and 60 dpi, with an increase in the number of CD8+ T cells being significant at 60 dpi and a significant increase in the CD8+/CD4+ ratio at 30 and 60 dpi (Figure S1).

**Figure 3:**
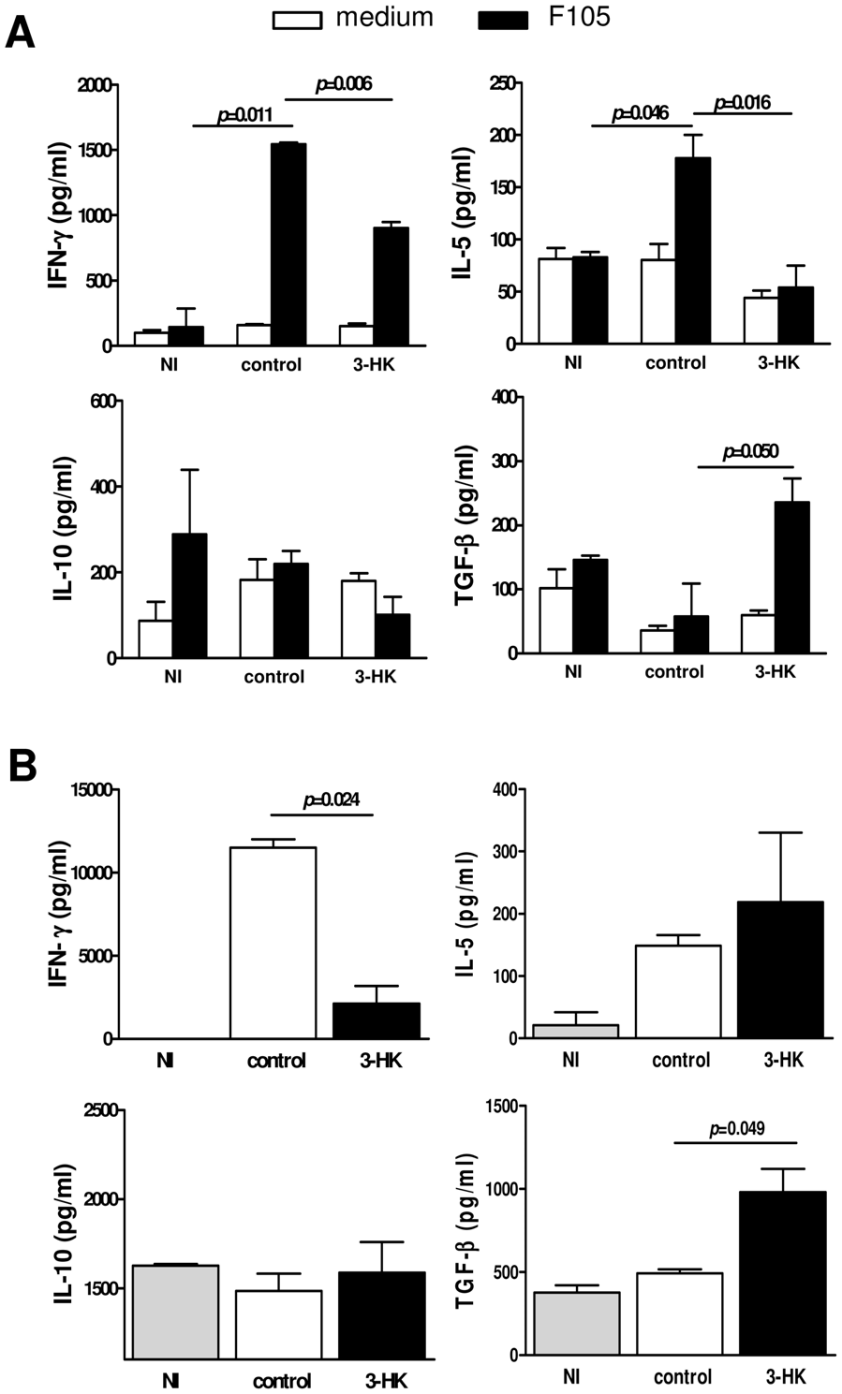
3-HK treatment modulates *T. cruzi* specific immune response during the acute phase of infection. (A) SMC from non-infected (NI) mice, 3-HK (n = 4) or control (n = 4) mice isolated at 16 dpi or from were cultured with or without 15 µg/ml of F105. After 72 h, supernatants were collected and the levels of secreted IFNγ, IL-5, IL-10 and TGF-β determined by ELISA. Bars represent the mean values ± SD from 4 mice/group. These results are from one of three similar experiments, all of which gave similar data. (B) Plasma IFN-γ, IL-5, IL-10 and TGF-β concentration in 3-HK (n = 4) and control (n = 4) 16-day *T. cruzi-*infected mice or non-infected (NI) mice. Data are shown as mean ± SD, n = 4 mice per group.

Then, we quantify the early cytokine response in plasma and culture supernatants of spleen mononuclear cells (SMC) cultured with parasite antigens at the peak of parasitemia (16 dpi). To carry this out, SMC from non-infected mice (NI) or 16 dpi mice, treated or not with 3-HK, were cultured with or without an extract of *T. cruzi* (F105) [19]. After 72 h, culture supernatants were collected and analyzed for IFN-γ, TNF, IL-1β, IL-12, IL-6, IL-4, IL-5, TGF-β and IL-10. As shown in Figure 3, SMC from control mice cultured with F105 produced significantly higher levels of Th1-type cytokine IFN-γ and of Th2-type cytokine IL-5 than SMC from NI mice. However, no differences between infected and NI mice were observed for TNF, IL-1β, IL-12, IL-6, IL-4, TGF-β or IL-10 cytokines (Figure 3A and not shown). In addition, SMC from 3-HK mice secreted significantly lower levels of INF-γ and IL-5 than SMC from control mice, with the levels of IL-5 being comparable to those secreted by SMC from NI mice (Figure 3A). Finally, SMC from 3-HK mice were able to secrete more than four times the amount of TGF-β produced by SMC from control mice (Figure 3A). In addition, lower levels of IFN-γ and higher levels of TGF-β were observed in plasma of 3-HK mice compared with control mice (Figure 3B). Taken together, these results indicate that the 3-HK treatment impaired at the acute phase of the infection the Th1- and Th2-type response against *T. cruzi,* while inducing cells able to secrete the immunoregulatory cytokine TGF-β.

**Figure 4:**
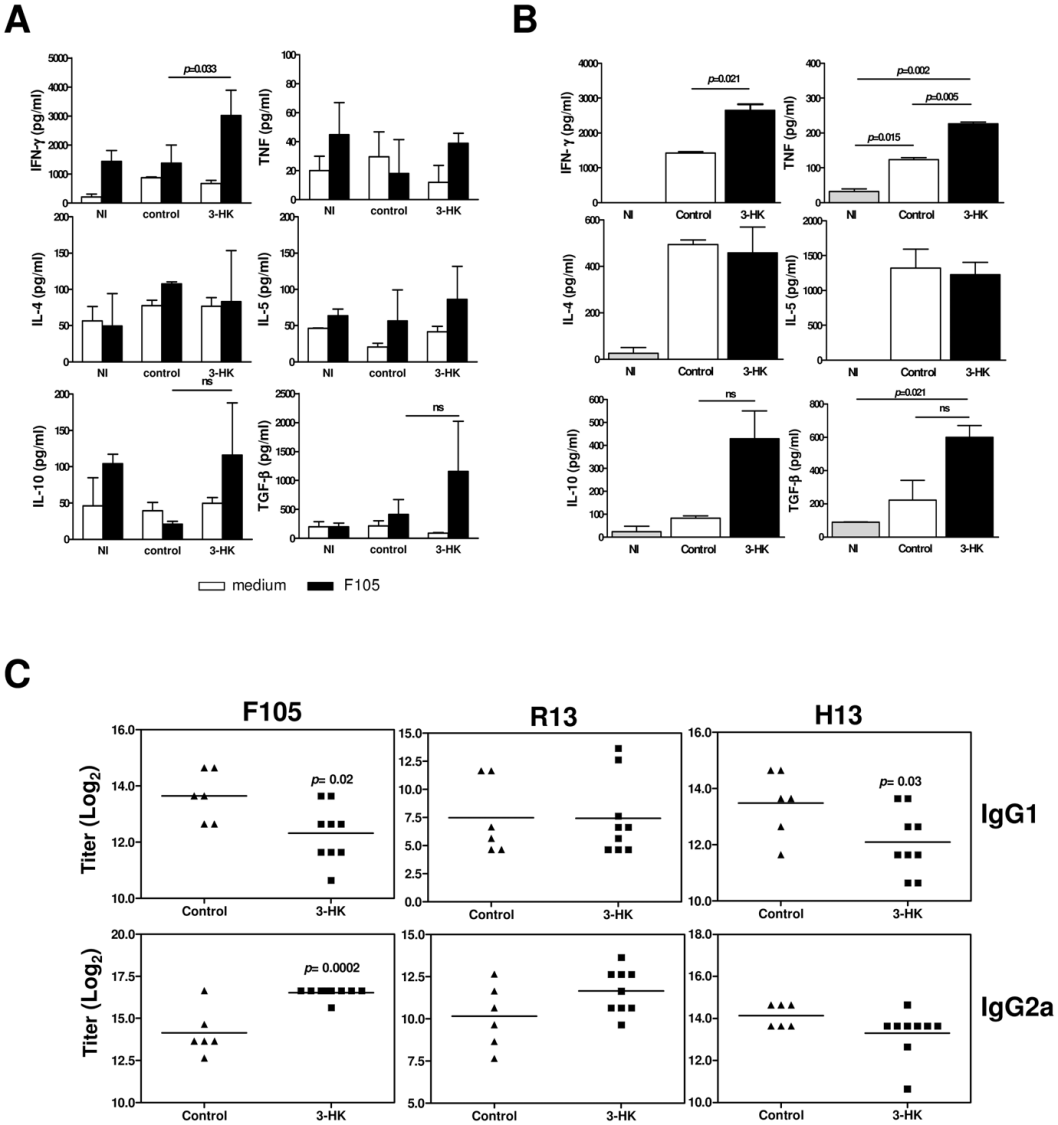
3-HK treatment induces a long-term Th1-type cellular and humoral immune response against *T. cruzi*. (A) SMC from 3-HK (n = 4) or control (n = 4) mice isolated at 60 dpi or from non-infected (NI) mice (n = 4) were cultured with or without 15 µg/ml of F105. After 72 h, supernatant were collected and the levels of secreted IFNγ, TNF, IL-4, IL-5, IL-10 and TGF-β determined by ELISA. Bars represent the mean values ± SD from 4 mice/group. These results are from one of three similar experiments, all of which gave similar data. (B) Plasma IFN-γ, TNF, IL-4, IL-5, IL-10 and TGF-β concentration in 3-HK (n = 4) and control (n = 4) 60-day *T. cruzi-*infected mice or non-infected (NI) mice. Data are shown as mean ± SD, n = 4 mice per group. (C) Sera from 3-HK or control mice at 60 dpi were assayed for detection of IgG1 and IgG2a isotypes of antibodies against F105, R13 and H13 by ELISA. Titrations were performed in duplicate, and the end point was expressed as the serum dilution with an optical density twice as high as those of the corresponding NI sera.

The cellular and humoral immune response developed in 3-HK mice during the chronic phase was then investigated. For that, SMC from NI mice or 60 dpi mice that were treated or not with 3-HK were cultured with F105. After 72 h, culture supernatants were collected and analyzed for Th1-type cytokines (IFN-γ and TNF), Th2-type cytokines (IL4 and IL5) and the regulatory cytokines IL-10 and TGF-β. SMC from 3-HK mice showed a long term Th1-type response with secretion of IFN-γ but not the Th2-type cytokines IL-4 or IL-5 (Figure 4A). Moreover, SMC from 3-HK mice showed a tendency to produce higher levels of the regulatory cytokines IL-10 and TGF-β than control mice (Figure 4A). In contrast, no differences between control and NI mice were observed for these cytokines, suggesting that at 60 dpi the cells able to respond to F105 antigen are absent in control mice (Figure 4A). In agreement, higher levels of the Th1-type cytokines IFN-γ and TNF and of the regulatory cytokines IL-10 and TGF-β were detected in sera from 3-HK mice compared with control mice (Figure 4B). These data show that 3-HK treatment was able to generate long-term *T. cruzi*-specific cells capable of secreting Th1-type and regulatory cytokines.

Then, we evaluated the effect of 3-HK treatment on the specific and autoreactive antibody response. To test antibodies against the parasite, F105 and the synthetic peptide R13 were used as antigens in ELISA assays. The R13 peptide sequence that corresponds to the C-terminal region of *T. cruzi* ribosomal P1 and P2 proteins is almost identical to the mammalian P1/P2 sequence, H13, except for one non-conservative amino acid substitution of an S by an E residue [20]. Since the incidence and levels of anti-R13 and anti-H13 antibodies are associated with the electrophysiological and histological alterations present in the heart of Chagas disease patients and *T. cruzi* infected or immunized mice [21]–[25], we used the synthetic peptide H13 to test the autorreactive antibody response. The quantification of IgG1 and IgG2a antibody isotypes against *T. cruzi* derived antigens (F105 and R13) and the autoantigen H13 revealed that, in agreement with the cytokine response against the parasite antigens, 3-HK mice showed a 4-fold increase in the levels of IgG2a and a 2-fold decrease in the levels of IgG1 antibodies against F105 compared with control mice, suggesting that 3-HK treatment skews the Th2-type antibody response (prevalent in BALB/c mice [26], [27]), towards a Th1-type antibody response (Figure 4C). In addition, the antibody response profile against R13 did not show significant differences between 3-HK and control mice. Interestingly, 3-HK treated mice showed more than a 2-fold decrease in the levels of IgG1 antibodies against the auto antigen H13 than control mice, while no significant differences were observed in the levels of IgG2a antibodies.

Summing up, these results suggest that the ability of 3-HK treatment to induce a healing response in *T. cruzi*-infected Balb/c mice was associated not only with a reduction in parasite numbers but also with a switch in the dominant Th2-type immune response observed in these animals.

### 3-HK treatment promotes Treg cell development

**Figure 5:**
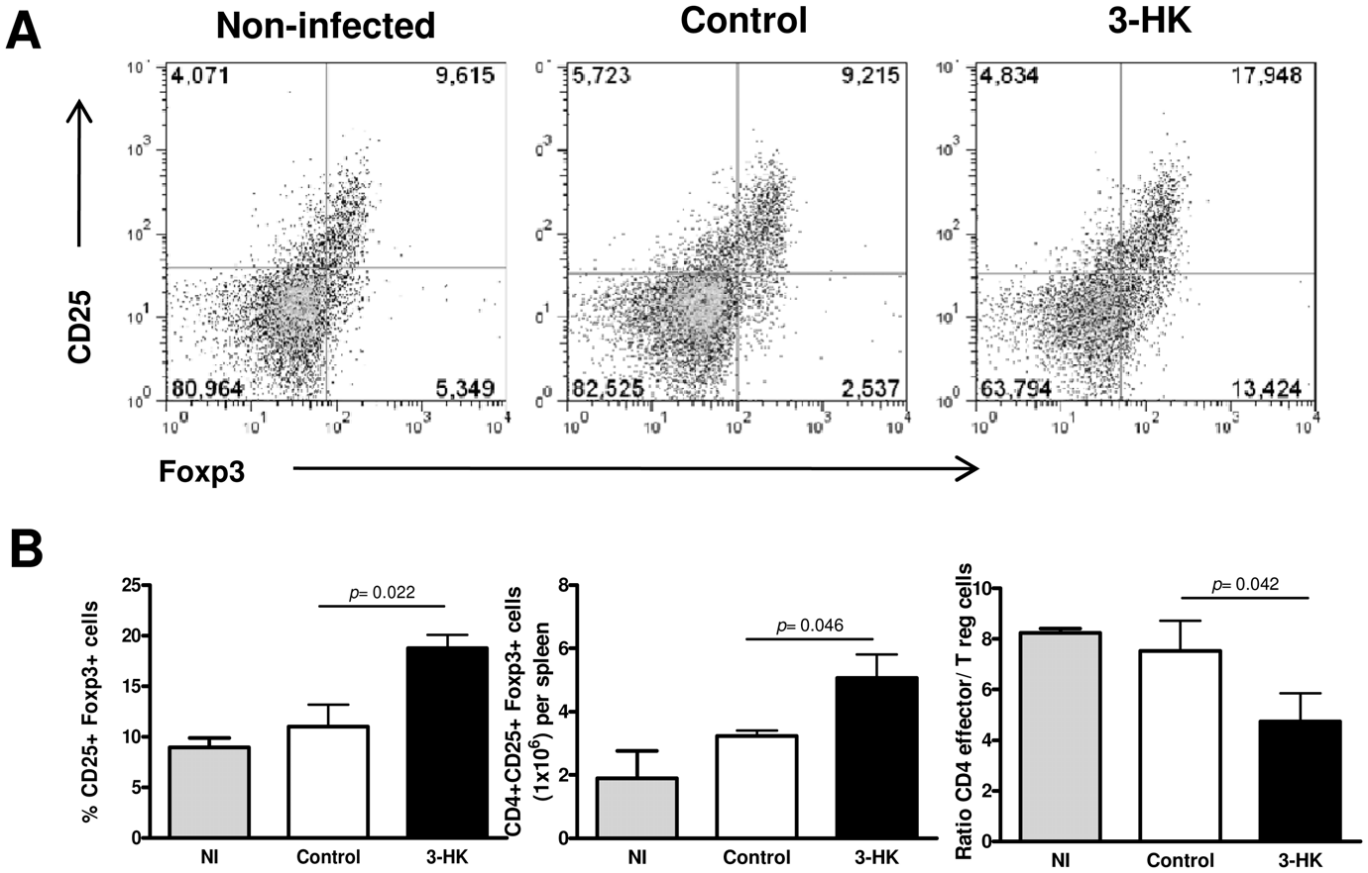
3-HK treatment promotes Treg cell development. Splenocytes from 3-HK (n = 4) or control (n = 4) mice isolated at 30 dpi were analyzed *ex viv*o by flow cytometry for the surface expression of CD4 and CD25 and the intracellular expression of Foxp3. Samples were collected using a FACScanto II flow cytometer and data were analyzed using FlowJo (Tree Star) software. (A) Plots from a representative animal with CD25 and Foxp3 expression on the CD4+ gated population are shown. The numbers represent the percentages of CD4+ cells expressing CD25+ and Foxp3+ in the live spleen population. (B) Percentage of CD4+cells expressing CD25+ and Foxp3+ (left panel), absolute number of CD4+CD25+Foxp3+ cells per spleen (middle panel) and the relative ratio of effector T cells (CD4+CD25-) per Treg cells (CD4+CD25+Foxp3+) at 30 dpi in NI, 3-HK and control groups. Bars represent the mean values ± SD from 4 mice/group. These results are from one of three similar experiments, all of which gave similar data.

Regulatory T cells (Treg) are important players in maintaining immune homeostasis and controlling excessive immune responses [28] which have been implicated in the suppression of Th1-mediated pathology or Th2 responses in a variety of settings [29]. Natural Treg cells that emerge from the thymus with self-specificity limit activation and expansion of autoreactive T cells in the periphery, whereas inducible Treg cells derive from conventional T cells that are antigen stimulated in the presence of mediators such as TGF-β and IL-10 [30]. The fact that SMC from acutely infected-3-HK mice produced little or no IFN-γ or IL-5 (Th1- and Th2-type cytokines respectively) but generated substantial amounts of TGF-β, together with increasing evidence of the involvement of IDO+ DC, IDO pathway catabolites and TGF-β in the induction of Treg cells, prompted us to investigate if 3-HK administration could result in Treg induction. We compared by flow cytometry the presence of CD4+CD25+Foxp3+ cells in spleen from 3-HK and control mice at different times pi (15, 30 and 60 dpi), and found that, similar to that observed in NI, ∼10% of CD4+ cells of the spleen from 30 day infected control mice were CD25+ Foxp3+ (Figure 5A, B), whereas after 3-HK treatment, the percentage of CD4+ cells in the spleen that were CD25+ Foxp3+ increased to ∼ 18%. This reflects a considerable expansion of the Treg population, because the absolute number of spleen cells was similar in both groups of mice (not shown). Accordingly, the ratio of effector CD4 T cells (CD4+CD25-Foxp3- cells) per regulatory T cells in the 3-HK group was 4.7 to 1 compared to 7.5 to 1 for control group (Figure 5B). We also detected the expansion of CD4+CD25+Foxp3+ at 15 dpi in one out of three experiments performed with four mice per group. Furthermore, we were unable to detect significant expansion of either CD4+CD25+ Foxp3+ cells at 60 dpi or CD4+CD25+CTLA-4+ or GITR+ cells at 15, 30 or 60 dpi (not shown).

### 3-HK induces Treg phenotype in T cells from *T. cruzi* acutely infected mice

**Figure 6:**
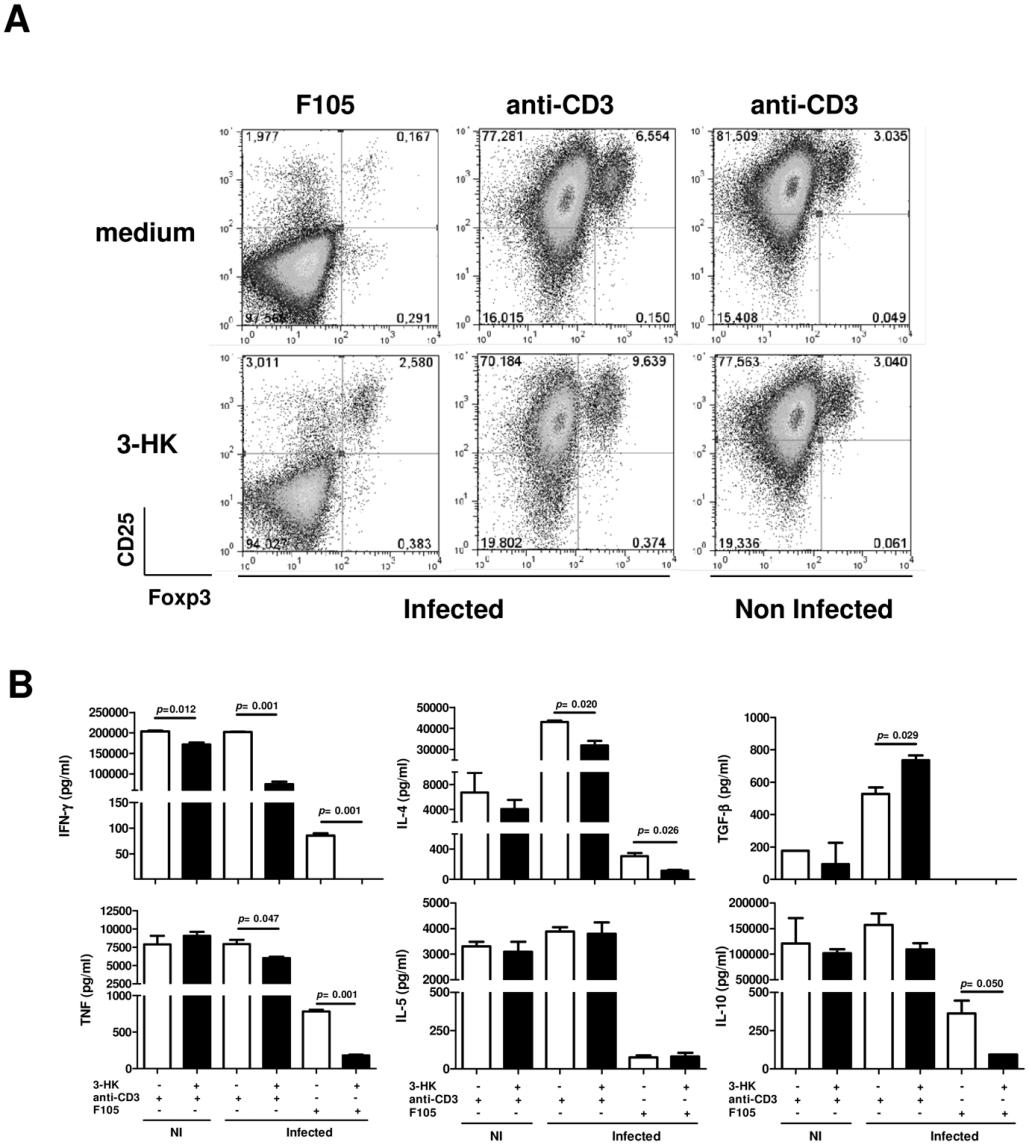
*In vitro*, 3-HK induces Treg phenotype in SMC from *T. cruzi* acutely infected mice. SMC from NI or 7 dpi mice were cultured with or without F105 (15 µg/ml) or anti-CD3 (1 µg/ml) in the presence or absence of 20 µM of 3-HK. (A) After 7 days of incubation, the cells were harvested and analyzed by combined surface expression of CD4+ and CD25+ and intracellular expression of Foxp3. Samples were collected using a FACScanto II flow cytometer and data were analyzed using FlowJo (Tree Star) software. A representative dot plot with CD25 and Foxp3 expression on the CD4+ gated population is shown. The numbers represent the percentages of CD4+CD25+Foxp3+ cells in the live population. Results show one experiment representative of three. (B) After 7 days of incubation, the secreted cytokines were determined in a cell culture supernatant after re-stimulation with plate-bound anti-CD3 (1 µg/ml) and soluble anti-CD28 (5 µg/ml) for 24 h. Data represent means ± SD of triplicate samples of one experiment representative of three.

To investigate whether 3-HK was able to induce *in vitro* the Treg phenotype, we decided to mimic *in vitro* the 3-HK administration *in vivo* in *T. cruzi* infected mice. To carry this out, SMC from 7 day-infected mice (a time point where the IDO activity has been induced by the infection [17]) were stimulated with F105 or anti-CD3 (to mimic the specific or polyclonal stimulation induced during the infection respectively) in the presence or absence of 20 µM of 3-HK for 1 week. In addition, we compared the effects of 3-HK on SMC from NI mice. The FACS analysis revealed that SMC from *T. cruzi*-infected mice, but not from NI mice, expanded the CD4+CD25+Foxp3+ cells when they were stimulated with *T. cruzi* antigens or anti-CD3 in the presence of 3-HK (Figure 6A). In addition, as we observed at 16 dpi during *in vi*vo 3-HK treatment, SMC from infected mice produced significantly lower levels of the Th1-type cytokines IFN-γ and TNF and also of the Th2-type cytokine IL-4 when they were stimulated with parasite specific antigen (F105) or polyclonally (anti-CD3) in the presence of 3-HK (Figure 6B). Moreover, 3-HK was able to suppress the antigen specific but not anti-CD3-induced secretion of IL-10 of SMC from infected mice. In addition, 3-HK was able to increase the secretion of TGF-β in cultures stimulated with anti-CD3 (Figure 6B). Finally, 3-HK was able to slightly suppress the secretion of IFN-γ of SMC from normal mice stimulated with anti-CD3 (Figure 6B).

## Discussion

Chagas disease is a tropical parasitic disease that is currently on the increase interest in non-endemic geographical areas such as the United States and Europe, mainly due to the population movement of infected people [2]. The fact that the parasite’s biological cycle in mammals includes the blood-circulating Tps and the replicating intracellular Am presents an extra challenge for those trying to develop new drugs for this disease because anti-*T. cruzi* drugs need to be able to cross the plasma membrane in order to be effective, in contrast with other trypanosome parasites, such as *Trypanosoma brucei*, which remain in the blood and so are potentially more accessible to drugs.

At present, Chagas’ disease desperately needs more treatment options. Benznidazole and Nifurtimox, the only existing current options, were introduced more than forty years ago and are not ideal, given that their side effects can be severe and can lead to resistance. Recently, we reported that 3-HK treatment of BALB/c mice lethally infected with *T. cruzi* is able to control the parasitemia and to improve the survival, with this effect being associated to the direct toxic effect of this compound on *T. cruzi* Am and Tps [17]. However, under the experimental conditions employed in those experiments, 3-HK treatment was only partially efficient in controlling the parasitemia and was not curative [17], although many authors consider that despite of their inability to eradicate the parasite, the drug-induced reduction of the parasite loads in infected tissues have positive effects on Chagas disease clinical evolution by reducing the severity of the associated inflammatory processes [31]–[34].

In the present study, we evaluated the effect of 3-HK on the subsequent chronic Chagas disease by using this treatment for 5 days during the acute phase of the infection of BALB/c mice that had been infected with a parasite dose that allows development of the acute infection and progression to the chronic phase. Once more, although for the lower infective dose used, 3-HK treatment was partially effective in controlling the parasitemia and the parasite load in target tissues in BALB/c and C57BL/6 mice (not shown); it was not curative for any of the 3-HK doses used. Moreover, after i.p. administration of 1, 5, 10, 50, 100 or 500 mg/kg/day, the effect on parasitemia did not increase proportionally with dose, indicating that saturation had occurred in the absorption kinetics of 3-HK.

Because 3-HK treatment is unable to eradicate the parasite, together with the known proapoptotic and immunoregulatory properties of 3-HK and their downstream catabolites, the outcome of this treatment on the development of the chronic phase of Chagas disease is difficult to predict. Nevertheless, in the present study we demonstrated that 3-HK treatment can be effective in the control of chronic Chagas disease development with functional and histological studies performed at day 60 pi showing that 3-HK treatment was effective in preventing or at least reducing the severity of cardiac complications, which are the major cause of death in chronic Chagas disease patients. One possible explanation for this result could be that reducing parasite burden made 3-HK treatment more effective in controlling Chagas disease development. However, differential susceptibility to *T. cruzi* infection and Chagas disease development does not seem to be only associated to parasite burden but also to a finely tuned balance between the appropriate and inappropriate induction of pro- and anti-inflammatory mediators [35]. Thus, although C57BL/6 strain mount after *T. cruzi* infection a Th1-type response able to control more efficiently the acute infection than BALB/c strain (Th2-prone strain), they develop a progressive fatal disease after the infection with Tulahuen strain of *T. cruzi* which is associated with increased serum levels of pro-inflammatory cytokines, a less efficient specific IgG response but not with higher parasite burden [35]. On the other hand, it has been demonstrated that immunization protocols capable of inducing polarized Th1 but not Th2 responses are able to protect BALB/c mice against *T. cruzi* challenge [36]–[38], while the Th2-type antibody response in this strain of mice is associated with cardiac functional and structural abnormalities [39], [40].

When the effect of 3-HK treatment on the development of any particular cellular or humoral immune response able to contribute to the parasite clearance and/or the control of the inflammatory associated pathology was studied, 3-HK treatment impaired, during the acute phase of the infection, the parasite specific Th2-type immune response promoting the development of TGF-β secreting cells. These results are in agreement with those demonstrating that the Th2-type antibody response in this strain of mice is associated with cardiac functional and structural abnormalities [39], [40]. Certainly, 3-HK mice showed a shift from Th2-type to the Th1-type humoral response against *T. cruzi* antigens and autoantigens in concordance with a lower incidence and severity of the electrophysiological abnormalities found in these mice [21]–[25].

In addition, 3-HK mice showed, at the chronic phase of the infection, an increased CD8+/CD4+ cell ratio and a population of *T. cruzi*-specific cells capable of secreting Th1-type cytokines. The development of a stable, *T. cruzi*–specific, CD8^+^ T cell population with the characteristics of memory cells showing effector function and providing protective immunity upon rechallenge has been reported after Beznidazole-induced cure or after heterologous plasmid DNA prime- human adenovirus boost vaccination of C57BL/6 mice [41], [42]. Though, additional studies must be performed in order to well characterize the Th1-type cytokines producing cell population that emerge after 3-HK treatment. On the other hand, SMC from chronically infected 3-HK mice also showed a tendency to produce higher levels of the regulatory cytokines IL-10 and TGF-β than control mice when stimulated with parasite antigens, with similar cytokine profiles observed in plasma from chronically infected 3-HK mice, suggesting that the emerging Th1-type response could be controlled by these two regulatory cytokines in 3-HK mice.

Fallarino et al. [43] have reported that when CD4+ T cells are stimulated *in vitro* for 7 days in low Trp medium in the presence of Trp catabolites (or IDO+ DC) they produce little or no IFN-γ or IL-4. However, they produce TGF-β which is necessary for the induction of the Treg phenotype in T cells. Both these conditions, Trp catabolism and Trp catabolites, are present when cultures of SMC from *T. cruzi* infected mice (but not from NI mice) are *in vitro* activated in the presence of 3-HK (because of the known infection-induced up-regulated IDO activity [17]). These conditions are also present when 3-HK is administered in the acute phase of *in vivo* infection. In agreement, 3-HK treatment was able to promote TGF-β producing cells and Treg development which was observed in the spleen at day 30 pi and *in vitro* by stimulating SMC from *T. cruzi* infected mice. Several studies have painted a picture of TGF-β as being a largely immunosuppressive cytokine able to act on naive T cells by suppressing the proliferation [44], inhibiting Th1 cell polarization [45] and promoting the generation of inducible Treg cells [46], [47]. However it has been recently described that TGF-β enhances effector Th1 cell activation and promote its self regulation via IL-10 [48]. In agreement, Guiñazu et al. [27] demonstrated an association between high levels of IFN-γ accompanied with high levels of TGF-β and the absence of an autoimmune response in B6 mice (Th1-prone) immunized with a *T. cruzi* antigen. Thus, although it has been shown that TGF-β production during the acute phase of *T. cruzi* infection contributes to the intracellular parasite replication [49], [50], it is possible that because 3-HK directly controls the intracellular parasite replication, the early production of this immunomodulatory cytokine could be responsible for the Treg development and the Th1 and Th2 response suppression contributing to the early control of the inflammatory response.

The role of Treg cells during *T. cruzi* infection is complex, with divergent effects observed depending on the strain of mice (C57BL/6 vs BALB/c) and parasites. Thus, the relative amount of Treg not change significantly during the course of *T. cruzi* infection of C57BL/6 mice, with the depletion of these cells not appearing to play a major role in regulating effector responses during the acute or chronic phase of the infection [51], [52]. On the contrary, Mariano, et al. have reported the role of natural Treg cells in controlling the parasitism as well as the cardiac inflammation and regulatory cytokines production in BALB/c mice infected with Y strain of *T. cruzi* [53]. Therefore, these findings are in agreement with our study in which the 3-HK induced development of Treg was associated with the control of the inflammatory associated pathology in BALB/c mice.

Recently, it was demonstrated that the Aryl hydrocarbon receptor (Ahr), a ligand-activated transcription factor that mediates dioxin toxicity, is required to induce IDO in DC [54]. Moreover, Kyn, and to a lesser extent 3-HK, can activate this receptor present on naïve T cells with this activation leading to Ahr-dependent Treg generation, which has been demonstrated to be potentiated by TGF-β through Ahr up-regulation [55]. It is also plausible that the early TGF-β production could lead to Ahr up-regulation in DC and T cells, which might potentiate the effect of 3-HK (and other Trp catabolites produced during *T. cruzi* infection) on Treg and IDO induction. Experiments to investigate whether *T. cruzi* infection is able to modulate the Ahr expression on DC and T cells, and the role of Ahr expression on *T. cruzi*-induced IDO up-regulation and Treg generation are underway.

Taken together, the results presented here indicate that 3-HK treatment for *T. cruzi* acute infection ameliorates the clinical outcome of chronic Chagas disease through the control of the parasite replication and also by inducing immunomodulatory effects, thus creating a balance between the control and clearance of the infectious organism on the one hand and the prevention of the immune-mediated pathology on the other hand. Therefore, the successful treatment of *T. cruzi* infection may not require the perhaps unachievable goal of sterilizing treatment, but it might be equally important to provide an efficient control of the parasite burden and the immune response in order to avoid progression from *T. cruzi* infection to Chagas disease.

Finally, it is important to highlight that a putative *T. cruzi* kynureninase enzyme has been identified [56], which could be involved in avoiding the parasite own destruction by the host 3-HK, considering that as today there is no available knowledge on an alternative pathway for Trp catabolism or the existence of the Kyn pathway in this parasite. These facts suggest that the presence of this enzyme is one of the mechanisms by which *T. cruzi* subverts the host’s immune systems and therefore make the use of this compound highly attractive in combined therapies for the purpose of achieving a parasitological cure.

## Materials and Methods

### Mice and parasites

Female BALB/c mice, 6–8 wk old, were obtained from Comisión Nacional de Energía Atómica (CNEA; Buenos Aires, Argentina) and maintained according to the National Research Council's guide for the care and use of laboratory animals. The Tulahuen strain of *T. cruzi* was used, which was maintained by weekly intraperitoneal (ip) inoculations in mice. The studies were approved by the Institutional Animal care and Use Committee of the Faculty of Chemical Sciences, National University of Cordoba (Approval ID Res 459/09).

### 3-HK treatment and parasite load

Mice were infected with 500 Tps from *T. cruzi* and treated with 3-HK as described previously [57]. 3-HK was resuspended in 0.1 M PBS, with this vehicle also being employed as control. The levels of parasitemia were monitored in blood collected at different times post infection (pi) as previously described [17]. Five days post infection (dpi), mice were daily treated with 3-hydroxy-dl-kynurenine (3-HK) from Sigma-Aldrich (St. Louis, MO, USA) at 1, 5, 10, 50, 100 or 500 mg/kg/d for 5 consecutive days (dpi: 5–10) by the i.p. route. 3-HK was resuspended in 0.1 M PBS, with this vehicle also being employed as control. The levels of parasitemia were monitored in blood collected at different times post infection (pi) from the tails. For that, erythrocytes were lysed in a 0.87% ammonium chloride buffer, and viable Tps were counted in a Neubauer chamber. For determination of tissue parasitism, genomic DNA was purified from spleen, skeletal muscle and heart from 20 days infected mice using TRIzol reagent and following manufactureŕs instruction. Satellite DNA from *T. cruzi* (GenBank AY520036) was quantified by real time PCR using specific Custom Taqman Gene Expression Assay (Applied Biosystem) using the primer and probe sequences described by Piron et al [58]. A sample containing 200 ng of genomic DNA was amplified and considered positive for *T. cruzi* when the threshold cycle (CT) for the *T. cruzi* target was <45. Abundance of satellite DNA from *T. cruzi* was normalized to GAPDH abundance. (Taqman Rodent GAPDH Control Reagent, Applied Biosystem) and expressed as arbitrary units.

### Electrocardiographic studies

Double-blind electrocardiograms (ECG) were performed at 60 days pi with a conventional ECG recorder (Fukuda) run at 50 mm/s [59], [60]. The peripheral leads DI, DII, DIII, aVR, aVF, and aVL were utilized. As controls for the ECG studies, normal non-infected mice were used. The ECG registers were read taking into account the electrocardiographic pattern in normal and in *T. cruzi* infected mice previously described [61], [62]. All alterations suggestive of bundle branch blocks were recorded under the name of intraventricular blockade as suggested by Sadigursky & Andrade [61]. We also used their criteria to characterize the intraventricular conduction abnormalities and auricle–ventricle blocks.

### Histological studies

The heart and the skeletal muscle from the quadriceps were removed at different times pi, fixed in buffered 10% formalin (pH 7.0), and embedded in paraffin. Organs were cut with a microtome to obtain 5-µm slices, collecting 1 section every 100 µm of the tissue until 300 µm of each organ was sectioned, thus obtaining 3 of each organ belonging to 3 levels, which were then stained with hema-toxilyn/eosin (H/E). At least 25 fields from each section were checked for parasites and histopathology under an X40 objective of a photonic microscope (Axioskop; Carl Zeiss Vision, Oberkochen, Germany) in a blind study. Twelve random digital images per organ were taken at the 3 levels (4/level) with a Hitachi color camera (Hitachi, Tokyo, Japan) being employed to measure the number of amastigotes, the number of nests of the amastigotes, and the number of inflammatory infiltrates. Measurements were performed by the quantitative image analysis system Axiovision 3.0.6 (Carl Zeiss) as described previously [17]. In addition, 5-µm sections of tissues were prepared for Masson trichrome staining [63], which highlights type I collagen deposition, to assess fibrosis.

### Antigens

*T. cruzi* antigens (F105), were prepared from epimastigote (Tulahuen strain) harvested from cultures in monophasic medium [64]. The epimastigote homogenate was centrifuged at 105,000 X *g* as described previously [19], and the supernatant was used for ELISA and in vitro cultures. The synthetic peptide R13 (EEEDDDMGFGLFD) corresponding to the C-terminal domain of *T. cruzi* P1 and P2 ribosomal P proteins [65] and the synthetic peptide H13 (EESDDDMGFGLFD) corresponding to the C-terminal domain of mammalian P1 and P2 proteins [66], were synthesized and linked to BSA using glutaraldehyde by the Neosystem (Strasbourg, France).

### In vitro cultures

Spleen mononuclear cells (SMC) from normal (NI), infected non-treated (control) or infected 3-HK-treated (3-HK) mice were isolated at the indicated times pi. For cytokine determinations, SMC were cultured with or without F105 (15 µg/ml) at 4×10^6^ cells/ml in complete RPMI medium in 24-well plates for 72 h. Supernatants were collected and the secreted cytokines measured.

To determine the effect of 3-HK on Treg induction *in vitro*, SMC from NI or 7 dpi mice were cultured with or without F105 (15 µg/ml) or anti-CD3 (1 µg/ml, BD Pharmingen) in presence or absence of 20 µM of 3-HK at 2×10^6^ cells/ml of complete RPMI medium in 24-well plates. After culturing for 72 h, cultures stimulated with anti-CD3 were expanded with complete RPMI supplemented with 10 ng/ml of mouse rIL-2 (R&D Systems, Minneapolis, MN). One week after primary stimulation the presence of Treg cells was determined by FACS and the secreted cytokines were measured in culture supernatant, after re-stimulation with plate bound anti-CD3 (1 µg/ml, BD PharMingen) and soluble anti-CD28 (5 µg/ml, BD Pharmingen) for 24 h at 2×10^6^ cells/ml of complete RPMI medium in 48-well plates.

### Cytokine measurement

Cytokines were measured in cell culture supernatants by capture ELISA using antibodies and protocols suggested by the manufacturer (eBiociences). The detection limit of the ELISA determinations was 125 pg/ml for IFN-γ, 62 pg/ml for IL-6 and TGF-β; 31 pg/ml for IL-10, IL-4 and IL-5; and 15 pg/ml for TNF.

### Flow Cytometry

Treg cells phenotyping was carried out by staining for CD4 (PerCP-Cy5 anti–mouse CD4, RM4-5, BD PharMingen) and CD25 (APC anti–mouse CD25, PC61.5, eBiosciences, San Diego, CA, USA), followed by intracellular staining for Foxp3 (PE-labeled anti-Foxp3, FJK-16 s, eBiosciences) using BD PharMingen reagents according to the manufacturer's protocol. Isotype controls for each antibody (BD PharMingen) were also included. Samples were collected using a FACScanto II flow cytometer (BD Bioscience) and analyzed using FlowJo (Tree Star) software. Treg cells were identified by the phenotype of CD4+ CD25+Foxp3+.

### Antibody assays

The quantification of IgG1 and IgG2a antibody isotypes against R13, H13 and F105 was carried out as described [24]. Titrations were performed in duplicate and the end point was expressed as the serum dilution, with the optical density reading being twice that of the corresponding NI.

### Statistical analysis

The data are expressed as means ± SD. Each experiment was repeated at least twice. Statistical analysis was performed using the Student's t-test and ECG data were analyzed using ANOVA and Fisher exact test.

## Supporting Information

Figure S1 **Comparison of the total numbers of CD4+, CD8+ and CD8+/CD4+ cells ratio in the spleens of 3-HK and control mice at 16, 30 and 60 dpi.** Results are means ± SD of 4 mice/group. One representative of two experiments is shown.
(DOC)
