# Supplementary figures and images for: 3-Hydroxy Kynurenine Treatment Controls T. cruzi Replication and the Inflammatory Pathology Preventing the Clinical Symptoms of Chronic Chagas Disease
Source: PLoS One. 2011 Oct 19;6(10):e26550. doi: 10.1371/journal.pone.0026550 (PMC3197528; doi:10.1371/journal.pone.0026550)

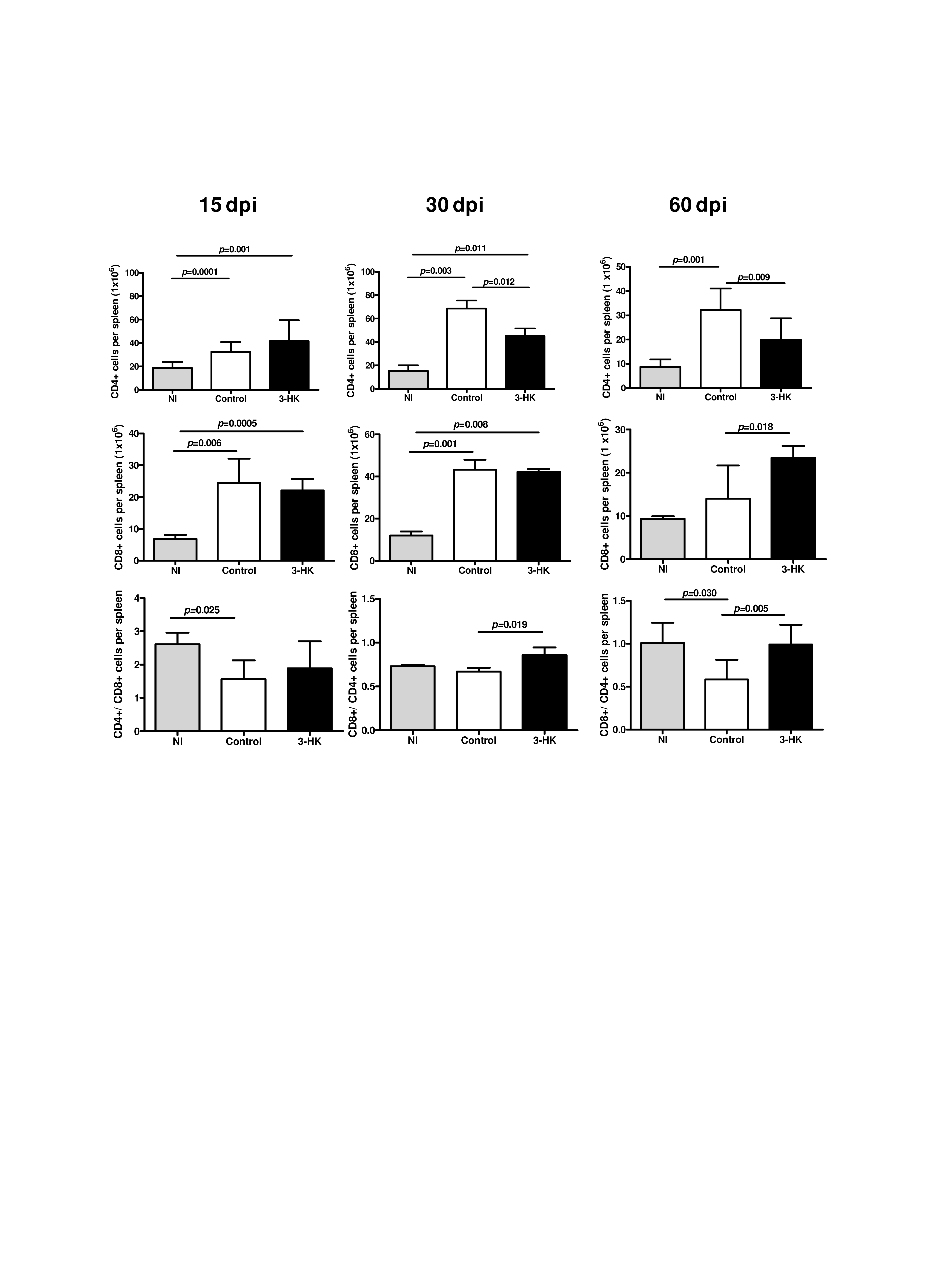

Supplement: Figure S1 — Comparison of the total numbers of CD4+, CD8+ and CD8+/CD4+ cells ratio in the spleens of 3-HK and control mice at 16, 30 and 60 dpi. Results are means ± SD of 4 mice/group. One representative of two experiments is shown. (DOC) [file pone.0026550.s001.doc]
